# CHARACTERISTICS OF MALADAPTIVE FALL RISK APPRAISAL AMONG OLDER ADULTS AGES 60 YEARS AND OLDER

**DOI:** 10.1093/geroni/igz038.1755

**Published:** 2019-11-08

**Authors:** Ladda Thiamwong, Boon Peng Ng

**Affiliations:** College of Nursing, University of Central Florida, Orlando, Florida, United States

## Abstract

Even though one-third of older adults have maladaptive fall risk appraisal (FRA), no studies has examined this discrepancy between perceived fall risk and physical fall risk among older adults in Thailand. We examined the characteristics of fall risk appraisal (FRA). 433 community-dwelling older adults were randomly selected from two provinces in Southern Thailand. Physical fall risk was assessed by a full tandem stand test and perceived fall risk was assessed by the Fall-Efficacy Scale International. We classified FRA into: 1) Rational FRA means low physical fall risk and low perceived fall risk; 2) Irrational FRA means low physical fall risk but high-perceived fall risk; 3) Congruent FRA means high physical fall risk and high-perceived fall risk; and 4) Incongruent FRA means high physical fall risk but low perceived fall risk. Irrational FRA and Incongruent FRA are a maladaptive FRA. About 60% of the participants had maladaptive FRA, which consisted of irrational FRA (57.3%) and incongruent FRA (2.3%). 20.8% were in rational FRA and 19.6% in congruent FRA. Among those with rational FRA, incongruent FRA, irrational FRA, and congruent FRA, 27.8%, 60%, 41.1%, and 74.1% reported having at least one fall in the past year, respectively. After covariate adjustment, participants in the congruent FRA group were 3.29 times more likely (p=0.006) to fall than those in rational FRA. High proportion of participants had maladaptive FRA so screening individuals with maladaptive FRA and prevent them to transition into the congruent FRA group is important efforts to mitigate health and economic burdens.

